# N-6 and N-3 Fatty Acid Cholesteryl Esters in Relation to Fatal CHD in a Dutch Adult Population: A Nested Case-Control Study and Meta-Analysis

**DOI:** 10.1371/journal.pone.0059408

**Published:** 2013-05-31

**Authors:** Janette de Goede, W. M. Monique Verschuren, Jolanda M. A. Boer, Lisa D. M. Verberne, Daan Kromhout, Johanna M. Geleijnse

**Affiliations:** 1 Division of Human Nutrition, Wageningen University, Wageningen, The Netherlands; 2 National Institute for Public Health and the Environment, Bilthoven, The Netherlands; University of Oxford, United Kingdom

## Abstract

**Background:**

Dietary polyunsaturated fatty acids (PUFA) are inversely related to coronary heart disease (CHD) in epidemiological studies. We examined the associations of plasma n-6 and n-3 PUFA in cholesteryl esters with fatal CHD in a nested case-control study. Additionally, we performed a dose-response meta-analysis of similar prospective studies on cholesteryl ester PUFA.

**Methods:**

We used data from two population-based cohort studies in Dutch adults aged 20–65y. Blood and data collection took place from 1987–1997 and subjects were followed for 8–19y. We identified 279 incident cases of fatal CHD and randomly selected 279 controls, matched on age, gender, and enrollment date. Odds ratios (OR) were calculated per standard deviation (SD) increase of cholesteryl ester PUFA.

**Results:**

After adjustment for confounders, the OR (95%CI) for fatal CHD per SD increase in plasma linoleic acid was 0.89 (0.74–1.06). Additional adjustment for plasma total cholesterol and systolic blood pressure attenuated this association (OR:0.95; 95%CI: 0.78–1.15). Arachidonic acid was not associated with fatal CHD (OR per SD:1.11; 95%CI: 0.92–1.35). The ORs (95%CI) for fatal CHD for an SD increase in n-3 PUFA were 0.92 (0.74–1.15) for alpha-linolenic acid and 1.06 (0.88–1.27) for EPA-DHA. In the meta-analysis, a 5% higher linoleic acid level was associated with a 9% lower risk (relative risk: 0.91; 95% CI: 0.84–0.98) of CHD. The other fatty acids were not associated with CHD.

**Conclusion:**

In this Dutch population, n-6 and n-3 PUFA in cholesteryl esters were not significantly related to fatal CHD. Our data, together with findings from previous prospective studies, support that linoleic acid in plasma cholesteryl is inversely associated with CHD.

## Introduction

Several reviews of prospective cohort studies and randomized trials suggest that the intake of n-6 and n-3 polyunsaturated fatty acids (PUFA) protect against coronary heart disease (CHD) [1], [2], [3], [4]. Linoleic acid, belonging to the n-6 PUFA family, is the most abundant PUFA in the diet and it is mainly obtained from vegetable oils, such as sunflower oil and soybean oil [2]. It is an essential fatty acid that can be elongated to arachidonic acid, which is also present in meat in small quantities [5], [6]. Alpha-linolenic acid is an essential fatty acid of the n-3 PUFA family and is present in soybean, canola, and flaxseed oil [2]. Alpha-linolenic acid can be elongated to eicosapentaenoic acid (EPA) and docosahexaenoic acid (DHA). Because these conversions takes place only to a limited extent (<8%),[7], [8], [9] EPA and DHA are mainly derived from the diet, through fish consumption [2].

Biomarkers of dietary intake are widely used in epidemiological studies [10], [11]. They are considered to provide a more accurate measure of intake than dietary records or questionnaire data, especially when the nutrient of interest varies widely within foods and food groups and when food composition tables are inaccurate for that specific nutrient [12]. Furthermore, biomarkers are not dependent on a person's ability to recall dietary intakes. Fatty acids can be measured as free fatty acids in serum (or plasma), as components of triglycerides, phospholipids, cholesteryl esters, erythrocyte membranes, platelets, or in adipose tissue from various sites [13]. Cholesteryl esters are found in plasma lipoproteins and reflect dietary intake of PUFA during the previous weeks [14], [15].

Harris *et al.* [16] performed a meta-analysis of 25 (nested) case-control studies and prospective cohort studies on tissue fatty acid composition and risk of CHD published until 2006. Harris *et al.* showed that long-chain n-3 PUFA tissue concentrations, especially DHA, were inversely associated with fatal CHD. However, in their meta-analysis crude data of PUFA levels were pooled, i.e. potential confounders were not taken into account. Furthermore, adipose tissue and various plasma and serum fractions were combined.

We investigated the associations of n-6 and n-3 PUFA, measured in plasma cholesteryl esters with the risk of fatal CHD in a prospective case-control study of Dutch adults, adjusted for confounders. Additionally, we performed a meta-analysis of nested case-control and cohort studies on plasma PUFA measured in cholesteryl esters in relation to CHD.

## Methods

### Ethical Statement

This research was performed in accordance with the ethical principles for medical research involving human subjects outlined in the Declaration of Helsinki. This research was approved by the Academic Hospital Leiden and the Medical Ethics Committees of TNO Prevention and Health, Leiden, The Netherlands. All participants gave written informed consent.

### Design and study populations

We conducted a nested case-control study using two similar consecutive Dutch population-based cohorts. The nested case-control design is considered an efficient alternative to a full-cohort analysis [17]. Baseline blood samples and information on lifestyle, and cardiovascular risk factors were collected in 35,475 subjects aged 20–59 years during 1987–1991 in the Monitoring Project on Cardiovascular Disease Risk Factors (subsequently referred to as MP-1) [18], [19] and in 22,654 subjects aged 20–65 years during 1993–1997 in the Monitoring Project on Risk Factors for Chronic Diseases (MORGEN Study; MP-2) [20]. For 7,754 participants who participated in both cohorts, we used the more recent MP-2 data. In addition, we excluded participants who did not provide informed consent for vital status follow-up and participants with a history of myocardial infarction (MI) or stroke at baseline, resulting in 26,987 participants in MP-1 and 21,335 participants in MP-2.

Vital status was checked through linkage with the national population register. Participants were followed for cause-specific mortality through linkage with Statistics Netherlands. Fatal CHD included fatal MI (I21, I22) and fatal cardiac arrest (CA; I46), according to the International Classification of Diseases (ICD-10, WHO). For causes of death coded until January 1, 1996, corresponding ICD-9 codes were used. Participants were followed until fatal CHD, death, date of loss-to-follow-up (predominantly because of emigration) or 1 January 2006, whichever came first.

All cases of fatal MI and fatal CA that occurred during follow-up (median 12.5 years, range 8–19 years) were identified. For each case (n = 232 in MP-1 and 69 in MP-2), one control from the same cohort was selected based on incidence density sampling to reduce the likelihood of biased results [21], [22]. Controls were selected from those persons under study who survived at least as long as the index case. A person was eligible to serve as a control for multiple cases at a given moment in time and could serve both as control and case. Cases were individually matched to controls on age (+/−0.5y), gender, and date of entry in the cohort (+/−0.5y). Plasma was available for 222 case-control pairs of MP-1 and 57 pairs of MP-2 and. In MP-1, five participants were selected as a control twice and four participants served both as a control and as a case. In MP-2, 1 participant was selected both as case and control.

### Measurement of n-3 PUFA in plasma cholesteryl esters

Participants donated non-fasting blood at baseline. EDTA-plasma of MP-1 was stored at −30°C and EDTA-plasma of MP-2 was stored at −80°C until analyzed. Fatty acids were measured in plasma cholesteryl esters by gas chromatography, as described previously [23]. In short, to isolate cholesteryl esters, lipids from EDTA plasma were dissolved and separated by solid phase extraction silica columns (Chrompack, Middelburg, The Netherlands). The fatty acids were identified by comparison with known standards (Nu-chek prep, Inc. Elysian, MN, USA). Fatty acids were expressed as mass percentages of total fatty acid methyl esters (g/100 g). A quality control plasma pool was analyzed in duplicate in each run. Coefficients of variation of the controls (intra and inter assay combined) ranged between 3 and 3.5 %. Laboratory technicians were blinded to the status of the samples. Cases and controls were randomly distributed over the runs.

### Data collection on risk factors

The baseline measurements were previously described in detail by Verschuren *et al*. [18], [20]. Body weight, height, and blood pressure were measured by trained research nurses. Hypertension was defined as a systolic blood pressure ≥140 mmHg, diastolic blood pressure ≥90 mmHg, or the use of blood pressure lowering medication. Non-fasting plasma was analyzed for total and high-density lipoprotein (HDL) cholesterol, and hypercholesterolemia was defined as plasma total cholesterol ≥6.5 mmol/l or use of cholesterol lowering medication. Self-administered questionnaires were used to assess the prevalence of diabetes, history of MI or stroke, medication use, parental history of MI, educational level, and cigarette smoking. Alcohol intake was calculated in glasses/d and was categorized as no intake, low to moderate intake (men ≤2 and women ≤1 glasses/d), or high intake (men>2 and women >1 glasses/d).

### Statistical analysis

In descriptive analyses, we compared the prevalence of risk factors and mean levels (±SD) of plasma fatty acids between cases and controls, stratified for cohort. The significance of differences in crude means or frequencies of risk factors were assessed by paired t-test for continuous variables and by the Wilcoxon signed-rank test for categorical variables. We used conditional logistic regression models to calculate odds ratios (OR) with 95% confidence intervals (95% CI) for the association of plasma levels of linoleic acid, arachidonic acid, alpha-linolenic acid, EPA, DHA, and EPA-DHA with fatal CHD. The analyses were repeated within the two separate cohorts. ORs and 95% CI for fatal CHD were calculated per SD increase in the plasma fatty acids, based on the distribution of controls.

In model 1, we adjusted for the matching factors age, gender, cohort, and enrollment date. In model 2, we additionally adjusted for current cigarette smoking (yes/no), body mass index (kg/m^2^), alcohol intake (no, low to moderate, or high), high educational level (completed higher vocational training or university). In model 3 we also adjusted for systolic blood pressure (mmHg) and plasma total cholesterol (mmol/l). Two-sided p-values <0.05 were considered to be statistically significant. Descriptive statistics and logistic regression analyses were performed with Statistical Analysis Software (SAS), version 9.2.

**Figure 1:**
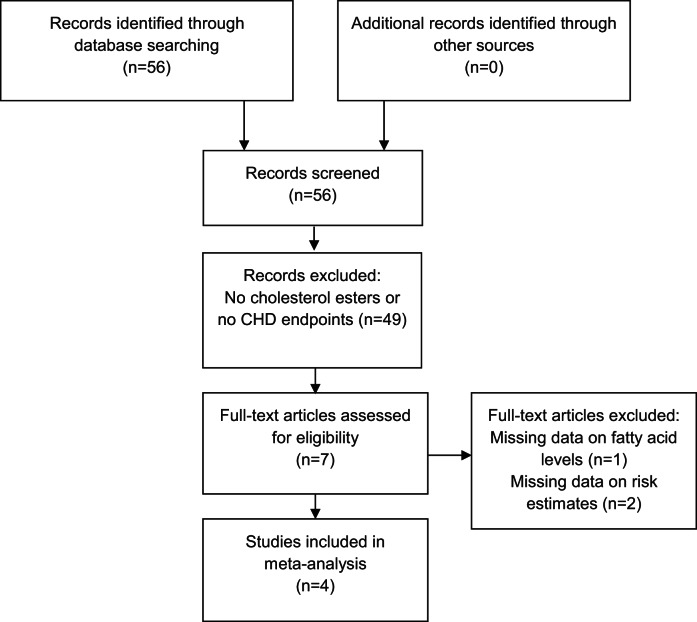
PRISMA 2009 Flow diagram meta-analysis.

We performed a dose response meta-analysis of all available published (searched with Pubmed) prospective studies (cohort studies or nested case-control studies) that measured cholesteryl ester PUFA status in relation to CHD risk including the estimates of the two separate cohorts of the current study. We identified five publications with relative risks on cholesteryl ester PUFA in relation to fatal and nonfatal CHD [24], [25], [26], [27], [28], of which one could not be used due to missing data on fatty acid levels [26] (Figure 1). In the original publication of Warensjö *et* *al* [28], the endpoint was fatal CVD. Data on CHD were kindly provided by the authors upon request. We used STATA version 11.0 (STAT Corp, College Station, TX) for meta-analyses using the METAN command. The generalized least-squares method for trend estimation of summarized dose-response data was used to calculate a relative risk for a certain unit of the exposure based on the Greenland and Longnecker method [29]. Each study was weighted by the inverse of its variance, including both the within and between study variance. Between-study heterogeneity was assessed via the I^2^ statistic, which expresses the percentage of variation attributable to between-study heterogeneity [30]. Random effects pooling were conducted according to DerSimonian and Laird [31]. We visualized and summarized the associations between different PUFA and CHD outcomes in forest plots.

## Results

### Nested cases-control study of plasma cholesteryl fatty acids and fatal CHD

Cases comprised 235 fatal MI (187 from MP-1 and 48 from MP-2) and 44 cardiac arrest events (35 from MP-1 and 9 from MP-2). Cases and matched controls from MP-2 were on average around 51 years old and 79% was male. Compared to MP-2, cases and controls from MP-1 had a similar age, but consisted of fewer males (70%). In both cohorts, cases had a higher body mass index, smoked more, more often used anti-hypertensive medication, and had higher blood pressure levels than controls. Cases of MP-1 also had higher plasma total cholesterol levels compared to controls (Table 1).

**Table 1:** Baseline characteristics of 279 fatal coronary heart disease cases and 279 matched controls.^*,†^

	MP-1			MP-2		
	Cases (n = 222)	Controls (n = 222)	P-value‡	Cases (n = 57)	Controls (n = 57)	P-value‡
Male gender, %	70	70	–	79	79	–
Age, y	50.5±7.4	50.5±7.5	–	51.7±7.1	51.8±7.2	–
Body mass index, kg/m^2^	26.9±4.7	26.0±3.9	0.02	27.7±4.8	26.2±3.8	0.07
Smoking, %						
Never	18	32	–	18	35	–
Former	21	30	–	26	39	–
Current	61	38	<0.0001	56	26	0.002
Alcohol consumption, %						
No intake	38	34	–	23	12	–
Low to moderate	34	36	–	44	67	–
High	27	30	0.29	33	21	0.90
High educational level, % §	7	14	0.008	21	18	0.82
Parental history of myocardial infarction, %	9.5	8.7	0.87	5.3	5.3	1.00
Diabetes mellitus, %	5.0	2.7	0.33	5.3	0	0.25
Systolic blood pressure, mm Hg	134.5±21.0	125.5±15.6	<0.0001	138.8±20.3	126.8±17.1	0.001
Diastolic blood pressure, mm Hg	83.4±12.4	78.6±9.7	<0.0001	85.6±12.1	79.7±9.9	0.002
Blood pressure lowering medication, %	14.9	9.0	0.06	19.3	10.5	0.23
Hypertension, %	46	28	<0.0001	56	33	0.002
Plasma total cholesterol, mmol/l ^||^	6.5±1.3	5.9±1.1	<0.0001	5.8±1.0	5.7±1.0	0.83
Plasma HDL-cholesterol, mmol/l ^||^	1.1±0.3	1.2±0.3	0.002	1.2 ±0.4	1.3±0.3	0.15
Cholesterol lowering medication, %	1.8	0.5	0.25	1.8	0	1.00
Hypercholesterolemia, %	42	27	0.001	26	19	0.42

Footnotes to Table 1.

* Values are means ± SD, unless indicated otherwise.

† Controls were matched on age, gender, cohort, and enrollment date.

‡ Paired t-test for linear values and Wilcoxon signed-rank test for proportions.

§ Completed higher vocational training or university.

|| Non-fasting.

Table 2 shows fatty acid levels for CHD cases and matched controls. The levels of linoleic acid, arachidonic acid, alpha-linolenic acid, and EPA-DHA were all lower in MP-1 as compared to MP-2. Linoleic acid values were (non-significantly) lower in cases compared to controls, although this was not statistically significant. The other fatty acid levels did not differ between cases and controls.

**Table 2:** Fatty acid proportions in plasma cholesteryl esters in 279 Dutch fatal CHD cases and 279 matched controls.^*,†^

Fatty acids (g/100 g) ‡		Cases	Controls	P-value §
*MP-1*		*N = 222*	*N = 222*	
Linoleic acid	C18∶2*n*-6	42.9±7.0	43.8±6.3	0.15
Arachidonic acid	C20∶4*n*-6	3.8±1.1	3.9±1.2	0.54
Alpha-linolenic acid	C18∶3*n*-3	0.39±0.13	0.38±0.14	0.56
EPA	C20∶5*n*-3	0.59±0.45	0.59±0.42	0.73
DHA	C22∶6*n*-3	0.33±0.16	0.33±0.15	0.59
EPA-DHA	C20∶5*n*-3+C22∶6*n*-3	0.92±0.57	0.91±0.54	0.996

*MP-2*		*N = 57*	*N = 57*	
Linoleic acid	C18∶2*n*-6	52.9±5.3	54.4±4.8	0.14
Arachidonic acid	C20∶4*n*-6	6.8±1.6	6.4±1.6	0.18
Alpha-linolenic acid	C18∶3*n*-3	0.51±0.15	0.51±0.14	0.95
EPA	C20v5*n*-3	0.93±0.64	0.89±0.57	0.88
DHA	C22∶6*n*-3	0.54±0.22	0.52±0.22	0.46
EPA-DHA	C20∶5*n*-3+C22∶6*n*-3	1.48±0.82	1.41±0.74	0.69

* Fatty acid levels are expressed as mass percentages (g/100 g).

† Controls were matched on age, gender, cohort, and enrollment date.

‡ Fatty acid levels are expressed as means ± SD.

§ Paired t-test, log transformed values were used for EPA, DHA, and EPA+DHA.

Cases and controls did not differ statistically in plasma n-6 and n-3 PUFA (Table 3). In the crude model, linoleic acid status was borderline significantly inversely associated with fatal CHD with an OR of 0.86 (95% CI: 0.74–1.00). In model 2, the OR (95% CI) for fatal CHD per SD increase in linoleic acid was 0.89 (0.74–1.06). Additional adjustment for plasma total cholesterol and systolic blood pressure (model 3) further attenuated the estimate. Plasma arachidonic acid was not associated with fatal CHD. The ORs (95% CI) for fatal CHD for an SD increase in n-3 PUFA were 0.92 (0.74–1.15) for plasma alpha-linolenic acid and 1.06 (0.88–1.27) for plasma EPA-DHA.

**Table 3:** Associations between plasma cholesteryl ester fatty acids and fatal CHD, matched by age, gender, cohort, and enrollment date.*

	Model 1 †	Model 2 ‡	Model 3 §
	OR (95% CI)	OR (95% CI)	OR (95% CI)
*Combined cohorts*	*N = 558*		
Linoleic acid	0.86 (0.74–1.00)	0.89 (0.74–1.06)	0.95 (0.78–1.15)
Arachidonic acid	1.00 (0.85–1.18)	1.06 (0.88–1.27)	1.11 (0.92–1.35)
Alpha-linolenic acid	1.05 (0.87–1.25)	0.97 (0.79–1.19)	0.92 (0.74–1.15)
EPA	1.02 (0.88–1.20)	1.07 (0.90–1.26)	1.04 (0.86–1.24)
DHA	1.02 (0.87–1.20)	1.09 (0.91–1.31)	1.12 (0.92–1.36)
EPA+DHA	1.03 (0.88–1.20)	1.08 (0.91–1.27)	1.06 (0.88–1.27)

*MP-1*	*N = 444*		
Linoleic acid	0.88 (0.75–1.05)	0.90 (0.74–1.10)	0.97 (0.78–1.21)
Arachidonic acid	0.95 (0.79–1.13)	1.02 (0.83–1.25)	1.08 (0.87–1.34)
Alpha-linolenic acid	1.06 (0.87–1.31)	1.01 (0.80–1.26)	0.93 (0.72–1.19)
EPA	1.01 (0.85–1.21)	1.06 (0.87–1.28)	1.02 (0.83–1.25)
DHA	1.00 (0.84–1.20)	1.07 (0.88–1.30)	1.11 (0.89–1.37)
EPA+DHA	1.01 (0.85–1.20)	1.06 (0.88–1.29)	1.04 (0.85–1.28)

*MP-2*	*N = 114*		
Linoleic acid	0.77 (0.54–1.10)	0.80 (0.53–1.23)	0.83 (0.53–1.32)
Arachidonic acid	1.32 (0.88–1.96)	1.26 (0.79–2.03)	1.29 (0.78–2.12)
Alpha-linolenic acid	0.99 (0.68–1.45)	0.87 (0.56–1.37)	1.01 (0.61–1.70)
EPA	1.08 (0.76–1.51)	1.11 (0.72–1.70)	1.06 (0.65–1.74)
DHA	1.15 (0.77–1.71)	1.22 (0.72–2.05)	1.15 (0.65–2.05)
EPA+DHA	1.09 (0.77–1.55)	1.13 (0.73–1.77)	1.08 (0.65–1.80)

EPA: eicosapentaenoic acid, DHA: docosahexaenoic acid.

* Values are odds ratios (95% CI) per standard deviation increase, based on conditional logistic models.

† Crude model, matched for age, gender, cohort, and enrollment date.

‡ Additional adjustment for smoking, BMI, education level, alcohol intake.

§ Additional adjustment for systolic blood pressure, total cholesterol.

### Meta-analysis of prospective studies on cholesteryl ester PUFA in relation to CHD

**Figure 2:**
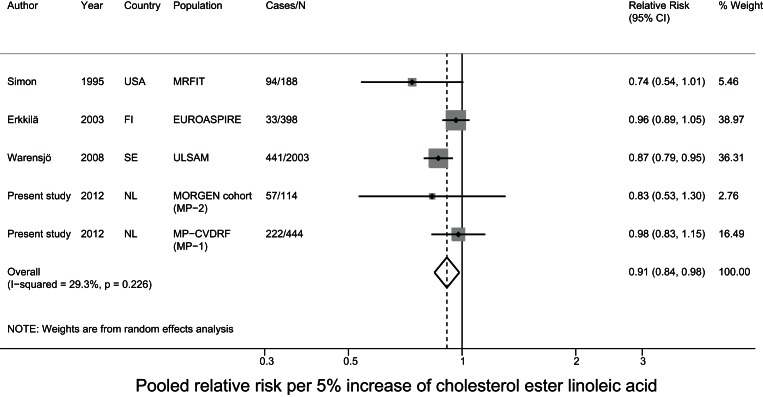
Pooled relative risk of cholesterol ester linoleic acid and CHD risk.

For the meta-analysis, we pooled the current data with results of two nested case-control studies from the USA[24], [25] and two cohort studies from Finland and Sweden on cholesteryl ester PUFA in relation to fatal and nonfatal CHD [27], [28]. The mean baseline age ranged from 50–60 years and the mean follow-up time ranged from 5–34 years between studies. Three studies comprised only men [24], [25], [28] and the other included men and women. For MP-1 and MP-2 we used the estimates of model 3, as that model was most comparable to the data of the other included studies. After pooling all studies, a 5% higher linoleic acid level was associated with a 9% lower risk (relative risk: 0.91; 95% CI: 0.84–0.98) of CHD. The other fatty acids were not associated with CHD. For DHA status we observed significant heterogeneity (p<0.001) (Figure 2–6). Exclusion of the study of Erkkilä *et al.,* which was the only study with coronary patients, resulted in a pooled OR (95% CI) of 1.09 (0.95–1.11) without heterogeneity (p = 0.49).

**Figure 3 pone-0059408-g003:**
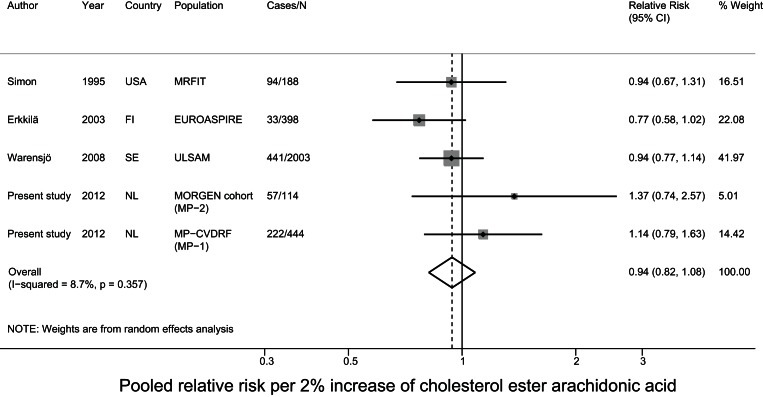
Pooled relative risk of cholesterol ester arachidonic acid and CHD risk.

**Figure 4 pone-0059408-g004:**
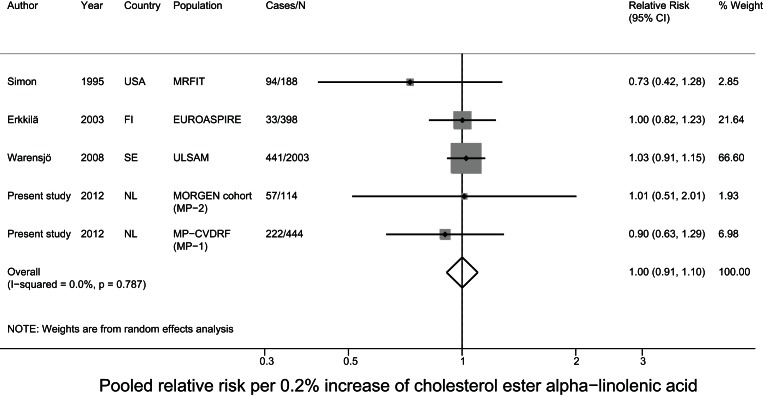
Pooled relative risk of cholesterol ester alpha-linolenic acid and CHD risk.

**Figure 5 pone-0059408-g005:**
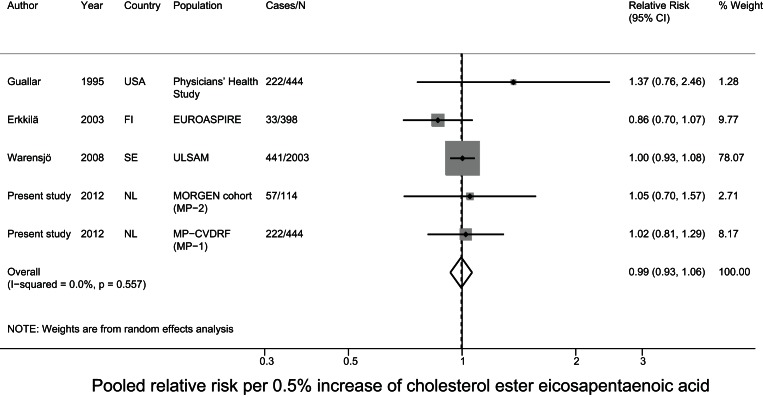
Pooled relative risk of cholesterol ester eicosapentaenoic acid and CHD risk.

**Figure 6:**
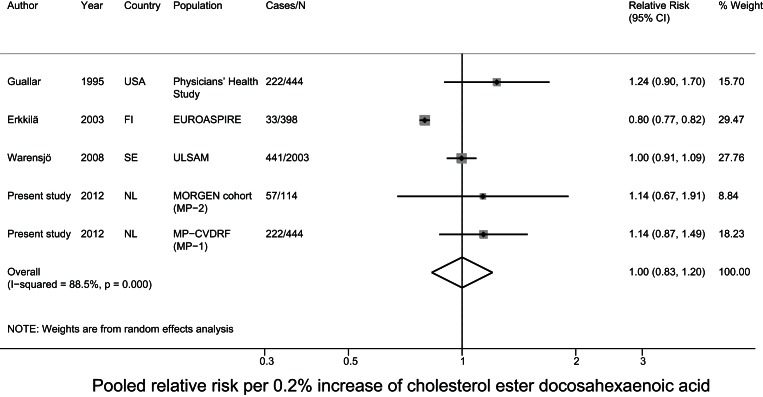
Pooled relative risk of cholesterol ester docosahexaenoic acid and CHD risk.

## Discussion

In a nested case-control study in Dutch adults we did not observe statistically significant associations between plasma cholesteryl ester linoleic acid levels and fatal CHD. When we pooled these data with those from similar prospective studies in a meta-analysis, a 5% higher linoleic acid level was related to a significant 9% lower CHD risk. Arachidonic acid and the n-3 PUFA alpha-linolenic acid, EPA, and DHA were not associated with CHD risk in the present study and in the meta-analysis.

A limitation of our study could be that the blood samples were stored for 18–23 years for MP-1 and 12–17 years for MP-2, which may have affected the quality of plasma fatty acids. However, storage up to 10 years at −80°C did not significantly influence serum cholesteryl ester fatty acid profiles in a recent validation study [32]. Although the n-6 and n-3 PUFA levels of the (older) MP-1 samples were considerably lower than those of the MP-2 samples, we do not expect that the values were differentially lower for cases compared to controls. The number of detected fatty acids (15–20) and the percentage of unknown fractions (rule of thumb <5%) were as expected for both cohorts. Furthermore, potential measurement error will have been random because the plasma samples of cases and controls were identically handled and analyzed in random order, and lab technicians were blinded for disease outcome. A strength of the present analysis was that we used two similar, large population-based cohort studies, with almost complete mortality follow-up.

The present nested case-control study did not show a statistically significant association between plasma cholesteryl ester linoleic acid levels and fatal CHD. However, a 5% higher linoleic acid level was related to a significant 9% lower CHD risk (OR: 0.91; 95% 0.84–0.98) in a meta-analysis in which we combined our findings with data from similar prospective studies. In the meta-analysis of Harris *et* *al*. [16], linoleic acid was not associated with CHD risk, based on a pooled estimate of seven prospective studies with various blood fractions. Plasma arachidonic acid did not predict CHD in our nested case-control study and meta-analysis, which was in agreement with Harris et al. [16]. In our nested case-control study and meta-analysis, we observed no association of cholesteryl ester alpha-linolenic acid or EPA-DHA with CHD, whereas Harris *et* *al*. [16] observed a borderline significantly lower alpha-linolenic acid status in CHD cases. Furthermore, DHA, but not EPA, was significantly inversely associated with CHD in the subgroup of prospective studies in the meta-analysis of Harris *et* *al*. [16].

The current meta-analysis and the one of Harris *et* *al*. [16] show different results, mainly for linoleic acid and DHA. Some differences in design could be responsible for this. Although Harris *et* *al*. combined data of a large number of studies, 16 out of the 25 studies had a classical case-control design (based on prevalent cases), which is more prone to reverse-causation and selection bias. Seven studies (case-control studies only) were based on adipose tissue samples. The other 18 used various blood fractions, such as phospholipids, cholesteryl esters, and erythrocytes, which could cause substantial heterogeneity in meta-analysis results. Finally, the analysis was based on crude PUFA levels. Potential confounding e.g. by body mass index and smoking, which appeared to be strong confounders in the present analysis, may partly explain discrepant results between the two meta-analyses.

Linoleic acid is by far the most important fatty acid in cholesteryl esters, followed by oleic acid, palmitic acid, and arachidonic acid [33]. In contrast, the concentrations of n-3 PUFA are very low. Therefore, in the n-3 PUFA, the variation between persons was probably small compared to the within-person variation. An American validation study reported that short and long-term reliability coefficients i.e. the ratio of between-person variance to total variance were >0.7 for cholesteryl ester linoleic acid, whereas these coefficients ranged between 0.4–0.5 for fatty acids that composed <1% of total cholesteryl ester fatty acids. The variance of the method was only <5% of the total variance [34]. A low between to within-person variation ratio will hamper finding significant associations between these fatty acids and fatal CHD. This is probably the reason why the associations between these PUFA and fatal CHD are inconsistent.

In conclusion, our findings in combination with those from other prospective studies support an inverse association between linoleic acid in plasma cholesteryl esters and CHD risk. For plasma cholesteryl ester levels of n-3 PUFA, however, no relations with CHD risk were found in our prospective study and meta-analysis, which raises concern regarding the validity of these biomarkers of intake for epidemiological studies.

## Supporting Information

Checklist S1 **PRISMA checklist.**
(DOC)

Figure S1 **PRISMA 2009 Flow Diagram.**
(DOC)
