# Supplementary material for: N-6 and N-3 Fatty Acid Cholesteryl Esters in Relation to Fatal CHD in a Dutch Adult Population: A Nested Case-Control Study and Meta-Analysis
Source: PLoS One. 2013 May 31;8(5):e59408. doi: 10.1371/journal.pone.0059408 (PMC3669344; doi:10.1371/journal.pone.0059408)
Supplement: Figure S1 — PRISMA 2009 Flow Diagram. (DOC) [file pone.0059408.s002.doc]

**PRISMA 2009 Flow Diagram**

Records identified through database searching

(n = 56 )

**Screening**

**Included**

**Eligibility**

**Identification**

Additional records identified through other sources

(n =0 )

Records after duplicates removed

(n = 56 )

Records screened

(n = 56 )

Records excluded

(n = 49 )

Full-text articles assessed for eligibility

(n = 7 )

Full-text articles excluded, with reasons

(n = 3 )

Studies included in qualitative synthesis

(n = not applicable)

Studies included in quantitative synthesis (meta-analysis)

(n = 4 )
